# Novel Soybean Variety Lacking Raffinose Synthase 2 Activity

**DOI:** 10.1021/acsomega.3c04585

**Published:** 2024-01-04

**Authors:** Miki H. Maeda, Kyoko Toda, Akito Kaga

**Affiliations:** † Research Center of Genetic Resources, National Agriculture and Food Research Organization (NARO), 2-1-2 Kannondai, Tsukuba, Ibaraki 305-8602, Japan; ‡ Institute of Crop Science, National Agriculture and Food Research Organization (NARO), 2-1-2 Kannondai, Tsukuba, Ibaraki 305-8518, Japan

## Abstract

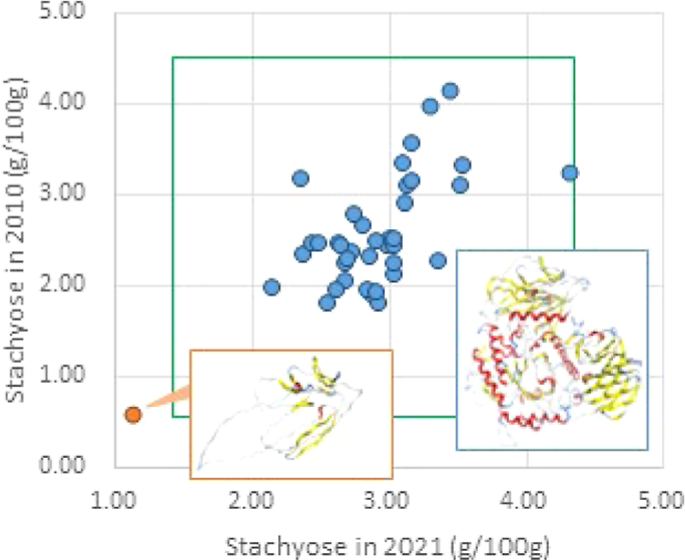

Variation in the raffinose family oligosaccharide (RFO) content in soybean is advantageous for livestock farming and health science. In this study, a soybean variety (GmJMC172) with a significantly low stachyose content in its seeds was identified in the NARO Genebank core collection. The results of the single-nucleotide polymorphism (SNP) analysis suggested that this phenomenon was related to a single-base deletion, inducing a frameshift mutation in raffinose synthase 2 (RS2), rather than the polymorphisms in the RS3, RS4, and stachyose synthase (STS) sequences. Differences in the enzymatic properties between the native RS2 and truncated RS2 were examined by using a three-dimensional model predicted using Alphafold2. In addition to revealing the missing active pocket in truncated RS2, the modeled structure explained the catalytic role of W331* and suggested a sufficient space to bind both sucrose and raffinose in the ligand-binding pocket. The soybean line, with seeds available from the NARO Genebank, could serve as breeding materials for manipulating the RFO content.

## Introduction

The NARO Genebank has been collecting soybean genetic resources since at least 1927. Currently (March 29, 2023), accessions of 8544 soybeans (*Glycine max*) and 2351 wild soybeans (*Glycine soja*) derived from domestic and overseas areas are available, and their information is searchable by keywords in our database.^1^ A mini-core collection was developed by rationalizing evaluation through geographical information and reducing redundancy^2^ to retain 100% of the gene diversity considering single-nucleotide polymorphism (SNP) variation, morphoagronomic trait variation, population structure, and geographic origin in all cultivated soybean accessions.^3^ This mini-core collection has been utilized for research on variation in traits such as shattering resistance associated with *Pdh1*,^4^ seed cesium concentration,^5^ and γ-glutamyl peptides, raffinose, and stachyose.^6^ Additionally, 198 accessions in the mini-core collection have undergone whole-genome Illumina resequencing to examine genetic diversity and facilitate the use of soybean genetic resources.^7^

Sucrose, raffinose, and stachyose are the primary soluble sugars found in soybean seeds. A standard cultivar, “Williams 82”, was found to contain 64.2 mg/g of sucrose, 12.6 mg/g of raffinose, and 41.0 mg/g of stachyose in its seeds.^8^ These soluble sugars are associated with the taste of soy-based foods such as tofu, miso, and natto,^6,9,10^ and their composition affects the fermentation process. Higher stachyose content results in a slower consumption rate, making it better suited for processing.^9,11^ Although raffinose and stachyose, both raffinose family oligosaccharide (RFO) members, are indigestible for animals and lower RFO content is preferable for feed purpose, they have also been reported to have a potential bifidogenic effect.^12,13^ Therefore, the composition of these three oligosaccharides is crucial for developing soybean cultivars tailored to specific applications.

**Figure 1:**
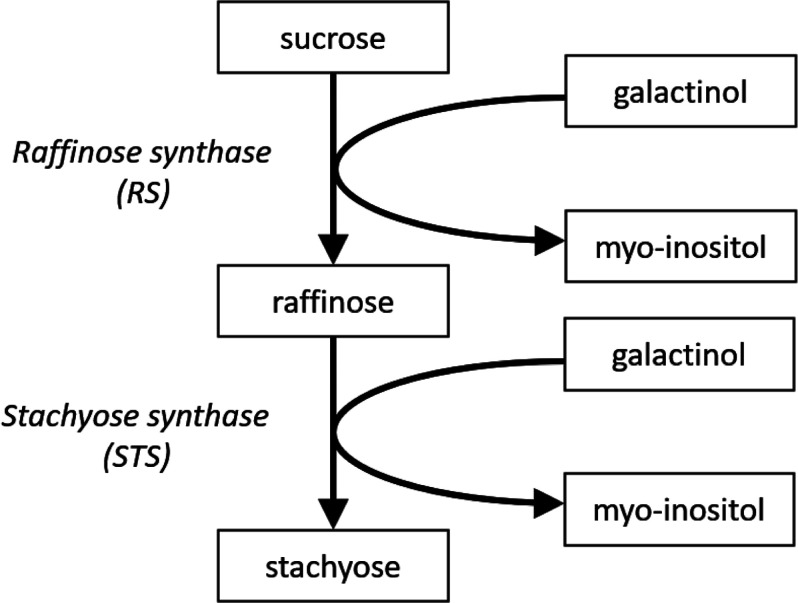
Biosynthesis of RFOs.

**Figure 2:**
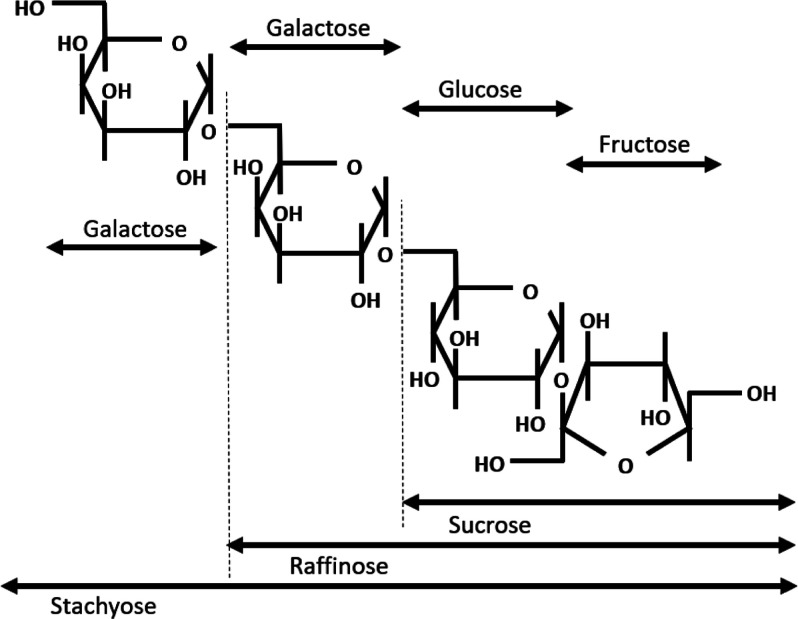
Structures of sucrose, raffinose, and stachyose.

Raffinose and stachyose are synthesized from sucrose through the sequential addition of galactose units by a set of distinct galactosyltransferases (Figures 1 and 2), and they play a key role in determining the ratio of the three primary oligosaccharides in soybean seeds. The enzymatic activities of raffinose synthase (RS) and stachyose synthase (STS) have been detected in seeds in the late developmental stage.^14^ In subsequent studies, soybean likely has three RS (EC 2.4.1.82) genes and one STS (EC 2.4.1.67) gene, namely, RS2 (Glyma.06G179200), RS3 (Glyma.05G003900), RS4 (Glyma.05G040300), and STS (Glyma.19G217700),^15−17^ although the roles of these enzyme homologues in raffinose and stachyose syntheses remain to be fully elucidated. However, mutations of the RS2 and STS genes have been reported to decrease the content of RFOs.^15,18,19^ To breed soybean with novel oligosaccharide characteristics and understand the roles of RS and STS, it would be useful to investigate the DNA sequence diversity in RS and STS as well as variations in the oligosaccharide content in our mini-core collections.

In this study, we present a novel soybean variety (GmJMC172) lacking RS2 activity. It was discovered from the soybean core collection of NARO Genebank^1^ as a variety with universally low stachyose content. Mean ± 3SD was used as a threshold to detect samples with significant difference from the other samples. Further, to investigate the mechanism underlying the activity decrease, we predicted the three-dimensional (3D) structure of RS2 and identified the amino acid residues comprising the ligand-binding pocket. Our analysis suggests the possible STS activity of RS2. We also discuss the importance of verifying predicted structures, especially where no experimentally obtained 3D-structure data is available. This structural verification process must be applicable to users attempting to apply the predicted 3D structures of proteins. Notably, the seeds of the analyzed varieties are available from the NARO Genebank.^1^

## Materials and Methods

### Preparation and Measurement of RFO Content in Soybean Seeds

Soybean mini-core collections, which were used for obtaining whole-genome resequencing data,^7^ were grown in our experimental field (Tsukuba Ibaraki, Japan) in 2010 and 2021. After harvest, the soybean seeds of each accession were air-dried for 6 months and then stored in a sealed polyethylene bag at 10 °C until use. The soybean lines used in this study are listed in Table 1. The seeds of all the analyzed accessions, including accessions GmJMC172, GmJMC030, and GmJMC158, can be obtained from the NARO Genebank.

**Table 1:** List of 39 Soybean Accessions Analyzed in This Study

accession of mini-core collection	name	original accession (JP number)
GmJMC008	KANAGAWA WASE	29621
GmJMC009	SHIZUNAIDAIZU	53267
GmWMC014	KLS 203	35506
GmWMC019	CHOUSENSHU(CHA)	28912
GmJMC021	OOYACHI 2	53264
GmWMC022	NEZUMI META	27956
GmJMC023	KUROGOYOU	28214
GmWMC027	KONGNAMUL KONG	29903
GmJMC030	KURODAIZU (AO HIGUU CHUU)	30135
GmJMC032	NATTOU KOTSUBU	29161
GmJMC034	MIYAGI SHIROME	27886
GmWMC036	MASSHOKUTOU (KOU 502)	27605
GmJMC041	DATE CHA MAME	28869
GmWMC042	MASSHOKUTOU (KOU 503)	27603
GmJMC050	FUKUI SHIRO	27948
GmJMC051	KURODAIZU (GEIHOKU)	227175
GmJMC054	ZAIRAI 51–6	29242
GmJMC055	SAKURAMAME	87833
GmJMC061	KOMAME	73030
GmWMC066	HEUKDAELIP	29803
GmJMC069	CHADAIZU	27890
GmWMC070	CHOYOUTOU	30071
GmWMC072	M 581	30258
GmWMC084	PEKING	28432
GmWMC086	ANTO SHOUKOKUTOU	35718
GmJMC092	KUROHIRA	27863
GmJMC099	AMAGI ZAIRAI 90D	76302
GmWMC103	SENYOUTOU	30072
GmJMC105	MAETSUE ZAIRAI 90B	76321
GmWMC107	HAKKA ZASHI	27543
GmWMC108	KARASUMAME	27584
GmJMC110	COL/TANBA/1989/ODAGAKI 2	147175
GmJMC114	COL/EHIME/1–2	227399
GmWMC118	OUDU	29882
GmJMC158	KUMAJI 1	28377
GmWMC159	COL/PAK/1989/IBPGR/2323(2)	74681
GmJMC172	TSURUSENGOKU	67989
GmJMC180	KOMUTA	29493
GmWMC187	KADI BHATTO	30419

**Figure 3:**
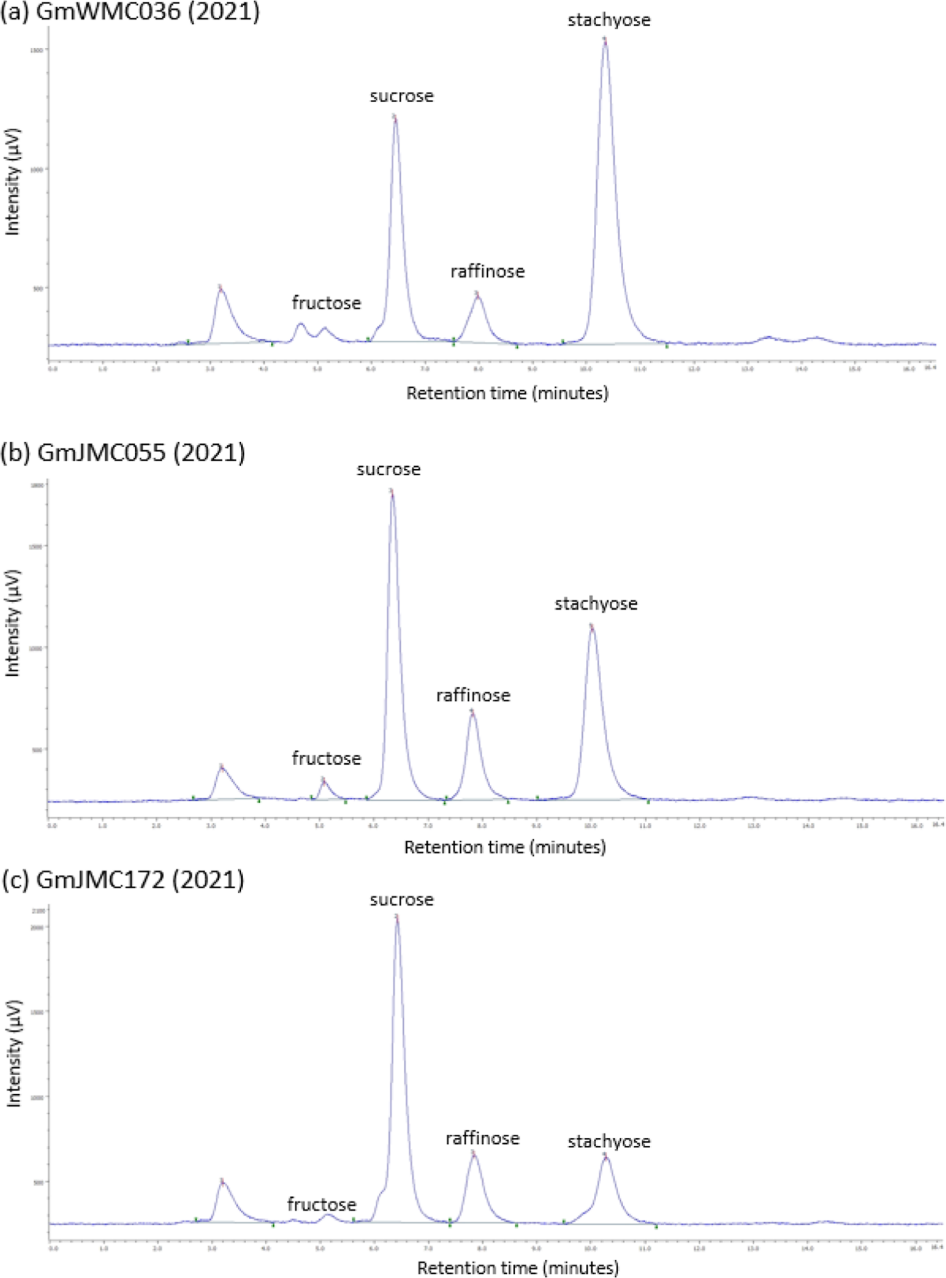
High-performance liquid chromatography (HPLC) charts of (a) GmWMC036, (b) GmJMC055, and (c) GmJMC172. HPLC was performed using a Shodex NH2P-50 4E column at 30 °C. The fluorescent responses of sugars were enhanced using a fluorescent developing reagent (50 mM guanidine-HCl, 1.5 mM periodic acid, 100 mM boric, and 125 mM KOH), excited at 325 nm, and detected at 420 nm. Sucrose, raffinose, and stachyose were identified based on the retention times of the standard compounds.

The soybean seeds were ground into a powder using a Retsch ZM 200 grinder (Retsch ZM 200, Retsch Düsseldorf, Germany) and stored at −28 °C. Each sample (1.5 g of the powder) was homogenized twice in 15 mL of 80% ethanol using a multibead shocker (Yasui Kikai, Osaka, Japan) with metal beads (Metal corn; Yasui Kikai). The resulting extracts were diluted with 80% ethanol to 45 mL and stored at −28 °C until use. The moisture content was calculated as the difference between the raw and dried weights after incubation of each sample powder at 105 °C for 24 h. The contents of sucrose, raffinose, and stachyose were determined as described by Masuda et al.^20^ with slight modification, as described in the Figure 3 legend.

### Computational Analysis

Statistical analyses were performed using R 4.2.2 and R-studio 2022.12.0 Build 353. Microsoft Excel was used to draw scatter plots. MOE 2020.0901 was utilized for visualization of molecules, modeling proteins and ligands, and docking analysis. The protein sequences of RS2 (I1KCD0) and STS (I1NBD9) were obtained from UniprotKB.^21^ The portion from the 30th to 781th residues of the I1KCD sequence was assigned as wild-type RS2 (WT-RS2). WT-RS2 sequence and the STS sequence were used to predict the 3D structure to prevent the interference of N-terminal peptide length during calculation because WT-RS2 and STS (I1NBD9) correspond from the first residue. Alphafold2^22^ version 2.2.0 was used to predict the protein structures from amino acids sequences, and one structure was selected from five candidates after manual verification and used for the further analysis. MOE-SiteFinder^23^ was applied to detect the ligand-binding pocket of the 3D structure, and the largest cleft was assigned as the ligand-binding pocket. To align WT-RS2 and STS sequences, 3D alignment employing predicted structures was performed using MOE.^23^ The complex structure of the ligand and enzyme was predicted using MOE-Dock^23^ with the AMBER10:EHT force field.^24,25^ Five docking poses were proposed via MOE-Dock, and the final model was selected based on its pocket environment, which had been reported previously in physiological studies.^15^

## Results

### Oligosaccharide Content in Soybean Seeds

Sucrose, raffinose, and stachyose contents in seeds were measured in 39 soybean accessions harvested in 2010 and 2021 (Table 2). Our high-performance liquid chromatography (HPLC) system was able to detect fructose, sucrose, raffinose, and stachyose, but no obvious peaks were observed for verbascose and longer oligosaccharides in any sample (Figure 3). Therefore, herein, “RFOs” in soybean seeds are defined as raffinose and stachyose. The peak ratio of sucrose, raffinose, and stachyose contents varied depending on the sample.

**Table 2:** Statistical Parameters of Sucrose, Raffinose, and Stachyose Contents According to Cultivation Year^a^

	year	data range (g/100 g)	mean (g/100 g)	SD (g/100 g)	Shapiro–Wilk test *p*-value	Wilcoxon test *p*-value
sucrose	2010	3.42–10.51	6.34	1.83	0.150	0.0775
	2021	3.51–9.04	6.02	1.41	0.277	
raffinose	2010	0.61–1.89	1.07	0.26	0.0697	6.78 × 10^–4^
	2021	0.54–1.21	0.92	0.18	0.204	
stachyose	2010	0.58–4.14	2.53	0.66	0.0475	1.28 × 10^–6^
	2021	1.22–4.47	3.10	0.51	0.00738	

a The underlined values indicate a statistical difference at a 95% significance level.

The mean and standard deviation (SD) of the sucrose content in the samples from 2010 and 2021 were 6.34 ± 1.83 and 6.02 ± 1.41 g/100 g, respectively (Table 2), which were not statistically different according to the Wilcoxon signed-rank test. However, both the distributions of raffinose and stachyose contents between 2010 and 2021 (Table 2) were statistically different (*P* = 6.78 × 10^–4^ and 1.28 × 10^–6^, respectively) according to the test. Specifically, the raffinose content was higher in 2010 than that in 2021, whereas the stachyose content was higher in 2021 than that in 2010.

**Figure 4:**
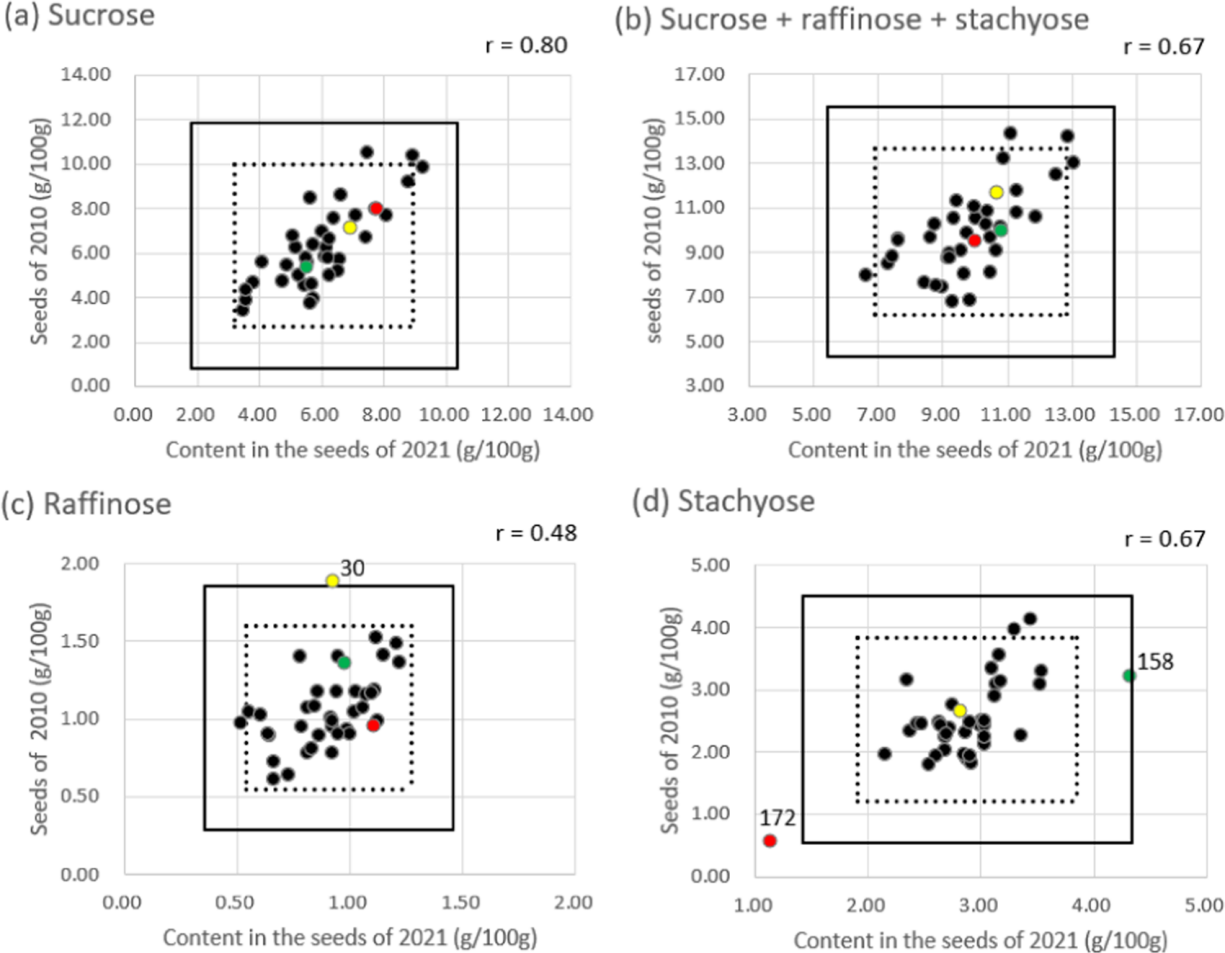
Saccharide content in soybean seeds cultured in 2010 and 2021. The correlation coefficients (r values) for 2010 and 2021 are displayed on each chart. The solid and dashed lines indicate the borders of the mean ± 3SD and mean ± 2SD of each distribution, respectively. The numbers near closed circles correspond to the accession numbers of the mini-core collection described in the text.

As shown in the scatter plots of Figure 4, GmJMC030, GmJMC158, and GmJMC172 were observed in the area approximately at or beyond the mean ± 3SD. Although the raffinose content of GmJMC030 exceeded the mean + 3SD in 2010, it was close to the mean value in 2021. Similarly, the stachyose content of GmJMC158 was greater than the mean + 3SD in 2021 but within the mean ± SD in 2010. These findings suggest that these two varieties have unique oligosaccharide synthesis properties that are influenced by some factors depending on the cultivation year. In contrast, GmJMC172 consistently exhibited much lower stachyose content than that in the other samples, indicating a unique event during the stachyose production process in GmJMC172.

### Q151fs Mutation of GmJMC172 Likely Causes the Loss of RS2 Activity

To elucidate the mechanism of the decrease in the stachyose content in GmJMC172, we analyzed all amino acid sequences of RS2, RS3, RS4, and STS for GmJMC172-specific mutations by SNP analysis. We identified a frameshift mutation at the 151th residue of RS2 as the “Q151fs” mutation, which is unique among all substituted amino acid positions of RS2, RS3, RS4, and STS (Table 3). To assess the effect of the Q151fs mutation on RS2 activity, we analyzed the protein structure.

**Table 3:** Amino Acid Substitutions Due to Single Nucleotide Polymorphisms in RSs and STS among 39 Soybean Accessions^a^

	STS	RS2							RS3			RS4	
	632	17	151	200	566	667	708	10	16	369	636	24	66
GmJMC008	M	T	Q	S	I	S	T	P	K	R	K	L	T
GmJMC009	M	T	Q	S	I	S	T	P	K	R	K	L	T
GmWMC014	K	T	Q	C	I	I	N	P	K	R	K	L	T
GmWMC019	K	T	Q	C	V	S	T	A	N	G	K	L	T
GmJMC021	M	T	Q	S	I	S	T	A	K	G	K	L	T
GmWMC022	M	T	Q	S	I	S	T	P	K	R	K	L	T
GmJMC023	M	T	Q	C	I	I	N	P	K	R	K	L	T
GmWMC027	M	T	Q	C	I	I	N	P	K	R	K	L	T
GmJMC030	M	T	Q	S	I	S	T	P	K	R	K	L	T
GmJMC032	M	T	Q	C	I	S	T	A	K	G	K	L	T
GmJMC034	M	T	Q	C	I	I	N	A	K	G	K	L	T
GmWMC036	K	T	Q	C	V	S	T	A	N	G	K	L	T
GmJMC041	M	T	Q	C	I	I	N	A	K	G	K	L	T
GmWMC042	K	T	Q	C	V	S	T	A	N	G	K	L	T
GmJMC050	M	T	Q	S	I	S	T	P	K	R	K	L	T
GmJMC051	M	T	Q	S	I	S	T	A	N	G	fs	L	T
GmJMC054	M	T	Q	C	I	I	N	A	K	G	K	L	T
GmJMC055	M	T	Q	S	I	S	T	P	K	R	K	L	T
GmJMC061	M	T	Q	S	I	S	T	P	K	R	fs	L	T
GmWMC066	M	T	Q	S	I	S	T	A	N	G	K	L	T
GmJMC069	M	T	Q	S	I	S	T	P	K	R	K	L	T
GmWMC070	M	I	Q	S	I	S	T	P	K	R	K	L	T
GmWMC072	M	T	Q	C	I	S	N	P	K	R	K	L	T
GmWMC084	M	T	Q	C	I	S	T	P	K	G	K	L	T
GmWMC086	M	T	Q	S	I	S	T	A	N	G	K	L	T
GmJMC092	M	T	Q	S	I	S	T	A	N	G	fs	L	T
GmJMC099	M	T	Q	C	I	I	N	A	N	G	K	L	T
GmWMC103	M	I	Q	S	I	S	T	A	K	G	K	L	T
GmJMC105	M	T	Q	S	I	S	T	P	K	G	K	L	T
GmWMC107	M	T	Q	C	I	S	T	P	K	R	K	L	T
GmWMC108	M	T	Q	C	I	S	T	P	K	R	K	P	T
GmJMC110	M	T	Q	C	I	S	T	A	K	G	K	L	T
GmJMC114	M	T	Q	C	I	I	N	P	K	R	K	L	T
GmWMC118	M	T	Q	S	I	S	T	A	N	G	K	L	T
GmJMC158	M	T	Q	C	I	I	N	A	N	G	K	L	T
GmWMC159	K	T	Q	C	V	S	T	A	N	G	K	P	T
GmJMC172	M	T	fs	C	I	I	N	P	K	R	K	L	T
GmJMC180	M	T	Q	C	I	S	T	A	N	G	K	L	T
GmWMC187	K	T	Q	C	V	S	T	A	N	G	K	L	T

a This mutation list was generated according to the data in the TASUKE database.^34^

**Figure 5:**
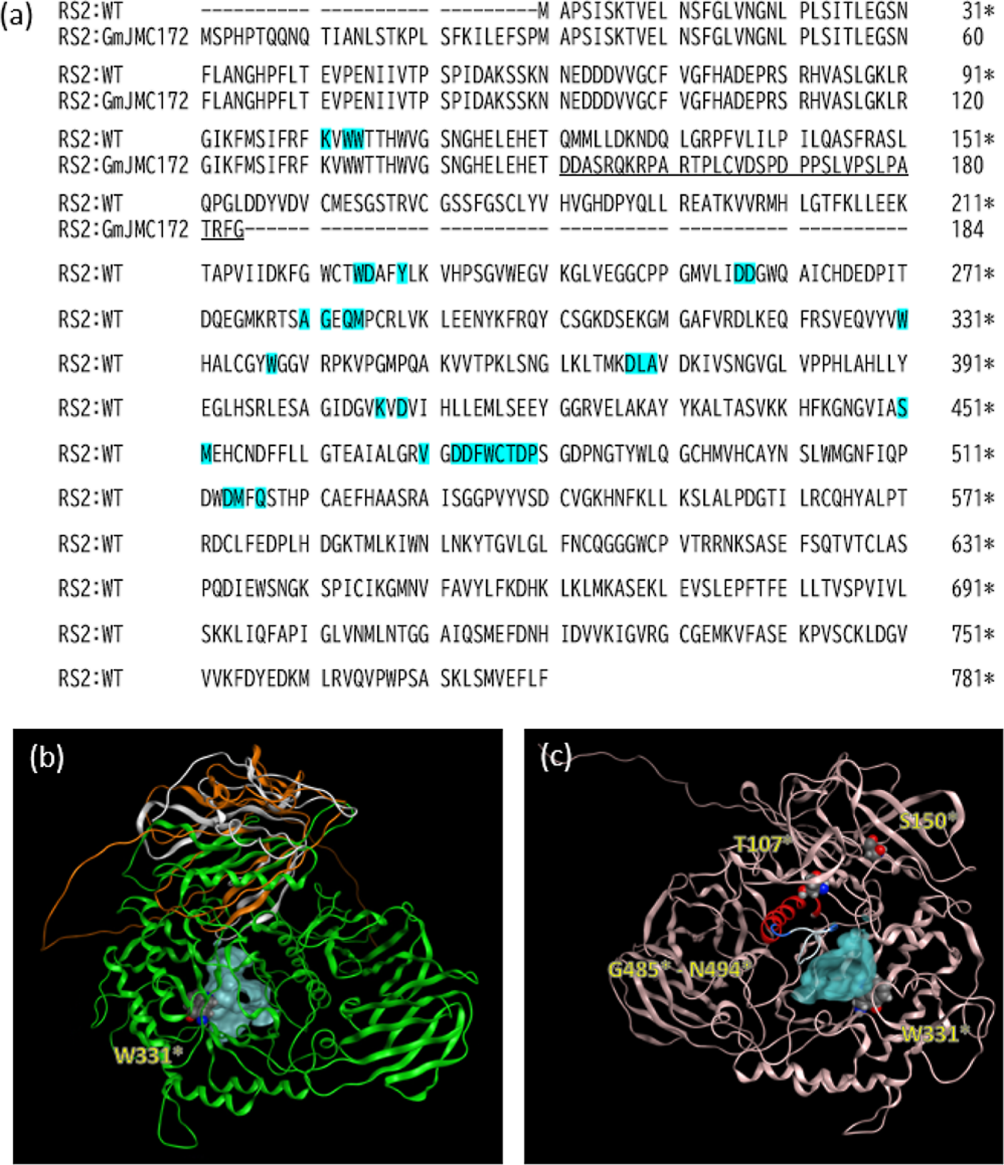
Comparison of WT-RS2 and GmJMC172-RS2. The noted residues are displayed as the space-filling model of CPK color with the names of yellow characters. (a) Pairwise alignment of WT-RS2 and GmJMC172-RS2 sequences. Because the residue numbers do not correspond between the two sequences, the residue numbers of WT are indicated as asterisks. The cyan-highlighted residues are found on the surface of the estimated active pocket in the Alphafold2-predicted modeled structure. The underlined residues indicate the chain following the frameshift mutation. (b) Superposed structures of predicted WT-RS2 (white/green tube) and GmJMC172-RS2 (orange tube). The green-colored structure is lacking in GmJMC172-RS2. The atoms drawn by the space-filling model indicate W331*. (c) Positions of T107*, S150*, and W331* on the predicted 3D structure. An α-helix from G485* to N494* fixed by T107* is colored red, and the continuing flexible loop consisting of the pocket is colored white (undefined secondary structure) and blue (turn).

As no 3D structures of RS2 were available in the Protein Data Bank, we employed Alphafold2 for prediction. The substrate-binding pocket, which included 30 residues, such as K131 (K102*), W133 (W104*), W134 (W105*), W254 (W225*), D255 (D226*), W258 (W229*), D286 (D257*), D287 (D258*), A310 (A281*), G311 (G282*), Q313 (Q284*), M314 (M285*), W360 (W331*), W367 (W338*), D397 (D368*), L398 (L369*), A399 (A370*), K436 (K407*), D438 (D409*), S480 (S451*), M481 (M452*), R499 (R470*), D502 (D473*), D503 (D474*), F504 (F475*), W505 (W476*), C506 (C477*), T507 (T478*), D508 (D479*), and P509 (P480*), was identified as the largest pocket in the modeled structure (cyan-highlighted residues of Figure 5a and cyan area in Figure 5b,c) because this pocket contained a tryptophan residue (W331*) that was previously reported to be an essential residue for decreasing the activity.^14^ An asterisk in the parentheses besides a residue shows the residue number in WT-RS2 described in the previous paper^15^ because the sequence is 29 residues shorter than the I1KCD0 sequence. To indicate these differences, the residue numbers of WT-RS2 are shown with asterisks in this article. The cyan surface depicted in Figure 5b consists of cyan-highlighted residues depicted in Figure 5a. As shown in Figure 5a, an alignment of the WT-RS2 and GmJMC172-RS2 sequences revealed a frameshift mutation at position Q151 (Q122*), likely resulting in a 155-amino acid protein in GmJMC172-RS2. Although three of the predicted pocket-surface residues were preserved, 90% of the residues in the pocket, including W331*, were lost in GmJMC172-RS2. To further examine the influence of the missing residues in the Q151 fs mutant, the lost residues are presented as a green ribbon in the 3D-structure of the wild-type RS2 (Figure 5b). This modeled structure clearly shows that the substrate-binding pocket is entirely absent from the native enzyme. This observation strongly supports the conclusion that the Q151 fs mutant in RS2 results in complete loss of enzyme activity.

### Verification of the Accuracy of Predicted Structures Using Alphafold2

To assess the accuracy of the predicted structures that have not been experimentally detected, we examined and confirmed our predicted 3D structure, which was deemed appropriate for the following reasons: (i) confirmation of three substitution positions (T107*, S150*, and W331*), as previously reported; (ii) identification of the insertion point of the distinct structure between RS2 and STS; and (iii) determination of the pocket shape of the galactinol–RS2 complex.

Figure 5c shows the T107*, S150*, and W331* positions. W331* is well-known to be associated with reduced enzyme activity.^15^ In our modeled structure, it forms part of the ligand-binding pocket and likely plays a crucial role in ligand–enzyme binding. As previously reported, the T107*I and S150*F substitutions have varying effects on the RS2 sequence:^26^ “the homozygous S150F line did not have an obvious oligosaccharide phenotype”, but “the T107I RS2 allele displayed a phenotype predicted for mutations in the soybean RS gene RS2.” These positions in our model are shown in Figure 5c. S150* is distanced from the active pocket and on a separate structural domain in the molecule, suggesting that the substitution is less associated with the enzyme activity. However, the side chain of T107* could form a hydrogen bond with the H497* side chain and fix the α-helix from G485* to N494* (Figure 5c). When the substitution from threonine to isoleucine occurs, the loss of hydrogen bonding would cause a change in the α-helix movement. Consequently, the flexibility in the turns and loops comprising V470*-N484* (part of the active pocket) alters the location of the α-helix. Consequently, this will cause a change in the substrate affinity.^26^

**Figure 6:**
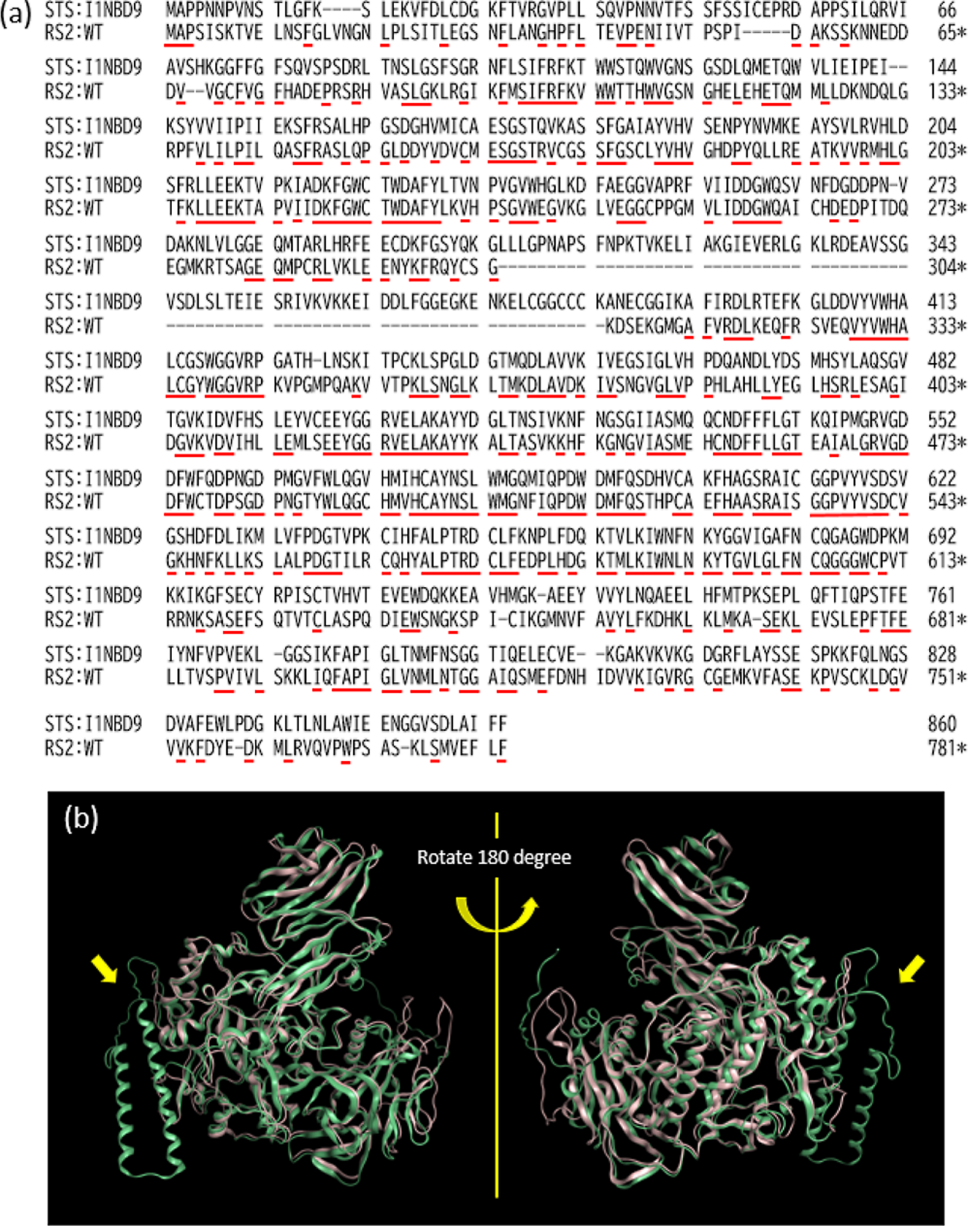
Comparison between WT-RS2 and STS. (a) Pairwise alignment of WT-RS2 and STS. The red lines under the sequences indicate conserved positions. (b) Superposed structures of the predicted WT-RS2 (tube of pale pink) and STS (light green). The yellow arrows indicate the two unique α-helices in the STS sequence.

As shown in Figure 6a, WT-RS2 and STS are well aligned because the conserved residues (pointed red circumflex accent) are distributed throughout. The inserted peptide, a major difference between RS2 and STS, was predicted in a reasonable location based on its 3D structure, i.e., the chain spanning residues 305–384 in the STS sequence. The insertion, predicted as two α-helices, turns, and loops, is unique in the STS sequence (Figure 6b). Thus, a secondary structure unit comprising two α-helices was incorporated through the connecting loops.

**Figure 7:**
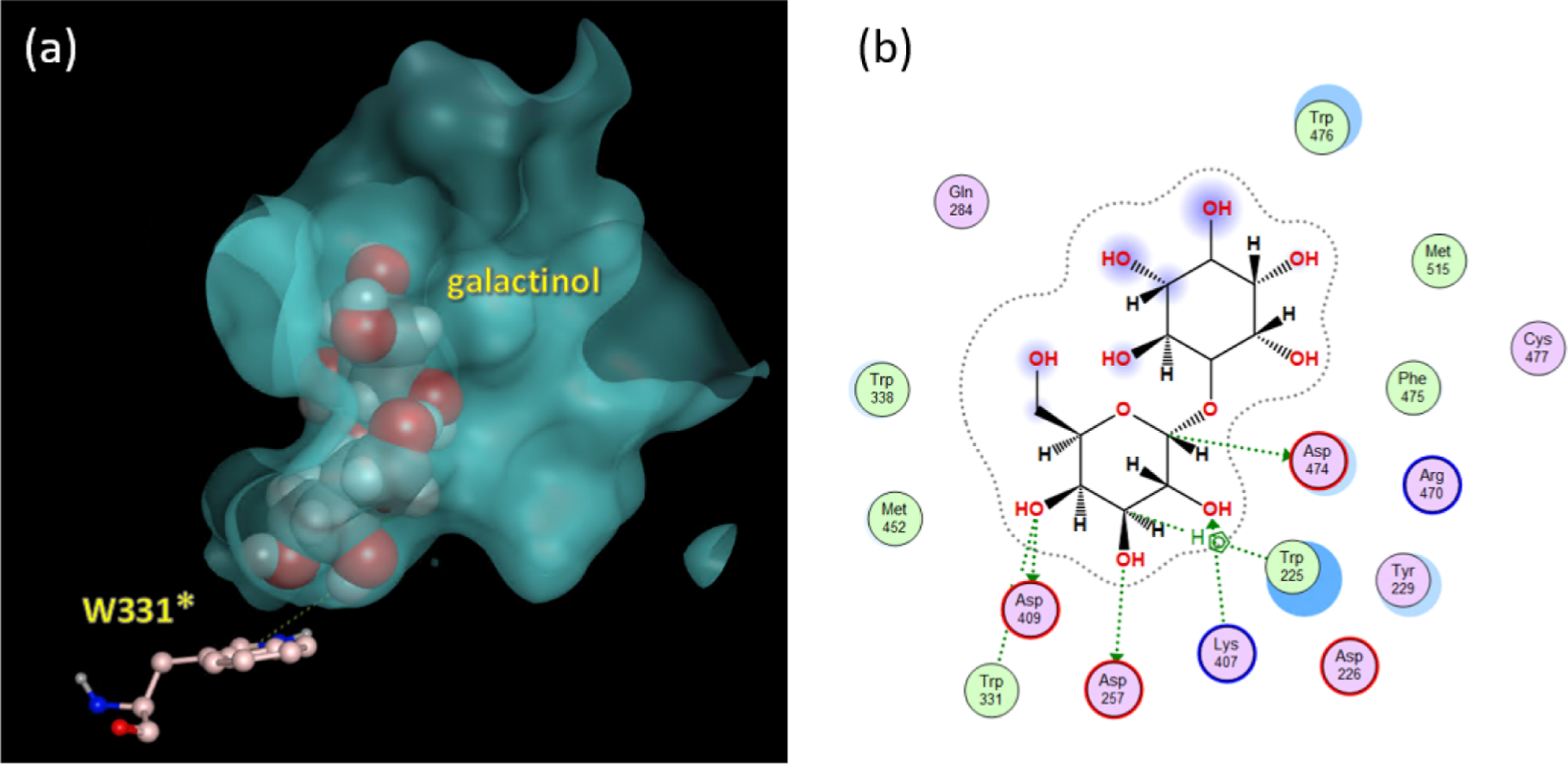
(a) Magnified view of the predicted RS2 pocket with galactinol (space-filling model) and W331* (pink ball-and-stick model). (b) Contact map of the modeled complex of galactinol and WT-RS2.

We constructed a modeled galactinol–RS2 complex structure, which yielded a docking pose that explains the hydrogen bond between W331* and the galactose residue of galactinol (Figure 7). In our modeled structure, the largest pocket is divided into two spaces, with galactinol located in one space near W331*.

### Discussion

Based on our measurement of the sugar content in soybean seeds, we identified GmJMC172 as an accession with markedly reduced stachyose content and the Q151fs mutation in the RS2 sequence. Given these findings, we now discuss the following: (i) the criteria for selecting varieties with distinctive traits based on the mean and SD, (ii) the differences in the sugar contents between years, and (iii) the functional estimation of RS2 based on a 3D structure model.

### Mean ± 3SD as a Threshold to Detect Statistically Significant Samples

If quantitative properties are measured for multiple samples, then it is necessary to select certain samples for further experimental analysis. To avoid arbitrary selection, statistical indices are employed for sample selection. In this section, we outline our approach.

Many natural phenomena are subject to various random events and often exhibit normal distribution. For example, the growth phenotype of a plant is influenced by numerous random factors, including gene mutations and climate conditions, and is therefore likely to follow a normal distribution. Here, we focused on the phenotype of the RFO content in soybean seeds. If random mutations are introduced into the genes of all accessions and the RFO content follows a normal distribution, special mutations should produce “remarkable” traits. Therefore, we used the mean and SD (herein, the SD of the population) to evaluate what constitutes a “remarkable” trait, regardless of the data set. Given a normal distribution, the probabilities of the ranges mean ± SD, mean ± 2SD, and mean ± 3SD were 68, 95, and 99%, respectively. Most samples fell within mean ± 2SD, so we used mean ± 2SD and mean ± 3SD as thresholds for detecting significant samples.

The scatter plots in Figure 4 show that the majority of samples fell within the mean ± 2SD (inside or around the broken line): only three samples (GmJMC030 for raffinose and GmJMC158 and GmJMC172 for stachyose) are located outside or near the solid line, which represents the mean ± 3SD threshold. Among these three samples, GmJMC172 consistently showed lower stachyose content across cultivation years, with a permanent defect of the RS2 molecule. Although the raffinose content of GmJMC030 fell within the mean ± 2SD in 2021, it was significantly higher in 2010. Similarly, the stachyose content of GmJMC158 was within the mean ± 2SD in 2010 but significantly higher in 2021. Therefore, it is necessary to consider alternative mechanisms for GmJMC172 and GmJMC030/GmJMC158. GmJMC172 likely has certain critical mutations regardless of cultivation or experimental conditions, whereas GmJMC030 and GmJMC158 may have mutations that alter expression for RSs and STS, respectively, depending on the conditions.

### Oligosaccharide Content and Its Causing Factors

GmJMC172 was found to have a stachyose content outside the mean ± 3SD threshold, regardless of the cultivation year (Figure 4d). This accession has a frameshift on the RS2 sequence, resulting in the loss of RS2 activity. The role of this frameshift mutation is further analyzed using molecular modeling in a later section. We also examined other samples with values outside the mean ± 2SD range but found no clear correlation between the SNPs and saccharide content. This suggests that only the mean ± 3SD threshold may detect critical changes on the gene. However, the raffinose content of GmJMC030 and the stachyose content of GmJMC158 varied between 2010 and 2021, suggesting that the samples in the range from mean ± 2SD to mean ± 3SD may have unique gene regulation mechanisms not found in standard accessions.

We assessed whether soybean seeds from 2010 and 2021 had significant differences in their saccharide contents, such as GmJMC030 and GmJMC158. We used the Wilcoxon test to compare the saccharide contents between the two years (Table 2). The sucrose contents were not statistically different, but the raffinose and stachyose contents were significantly different. This may reflect the number of metabolic reactions to produce or degradate a metabolits. If a metabolite was generated by a smaller number of pathways, one mutation should be much influenced to the content of it than that of another metabolites maintained by more pathways.

### Why Does a Lack of RS2 Activity Cause a Decrease in the Amount of Stachyose but Not That of Raffinose?

When the activity of a synthase is lost, we normally expect the amount of the enzyme product to decrease. However, as observed in this study, GmJMC172 did not decrease the raffinose content but instead decreased the stachyose content. It suggests that RS2 concerns stachyose synthesis.

The *rs2* mutant generated by Cao et al.^27^ showed reduction of both raffinose and stachyose. If the contents of the two oligosaccharides are linked, reduction of stachyose content could be interpreted by a decrease of raffinose caused by inactivated RS2. However, GmJMC172 showed only a decrease in the amount of stachyose but not raffinose. Hence, a new hypothesis that RS2 synthesizes both raffinose and stachyose is introduced, although the RS and STS synthesis activities were previously considered independent. Even then, the following question arises: why are the contents of both raffinose and stachyose not reduced simultaneously in GmJMC172?

A possible explanation is the contribution of other raffinose synthases such as RS3 and/or RS4. If raffinose synthesis is compensated by these synthases, then the content of raffinose may recover to the same level as that seen in GmJMC172. According to the report by Cao et al.,^27^ the *rs2* and *rs3* double mutant exhibited lower stachyose content than that of the *rs2* single mutant. This result hints at the possibility of RS3 contribution in raffinose and stachyose syntheses.

### Reliability of Modeled Structures Predicted Using Alphafold2

It has been reported that the prediction of 3D structures using Alphafold2 is accurate.^22^ However, the predicted structures should be trusted only after they match relevant biological experimental results. In this study, we verified our structures based on the biological data described above, and no contradictions were detected: First, our modeled structure of RS2 corroborates the experimental results of Dieking and Bilyeu.^26^ Second, an inset position between RS2 and STS is possible. It would be evolutionarily possible from a common ancestral protein of the RS2 type (a mechanism such as Go’s module shuffling^28^). Third, our subsequent analysis of the RS2–galactinol complex could explain the enzymatic mechanism of RS2. In the predicted structure, W331* is a part of the galactinol-binding subpocket. Regarding the enzymatic mechanism of glycosyltransferase,^29^ galactosylation proceeds either through a two-step reaction starting with the binding of galactinol and the enzyme or via an intermediate consisting of galactinol and sucrose. In both cases, the binding modes of galactinol connected to W331* in the pocket are necessary.

In conclusion, we deemed the predicted structures sufficiently accurate to discuss the limited coverage of the folding of the whole molecule, the 305th to 384th residues of STS, and the docking pose of galactinol.

### Estimation of the Docking Pose and Possible Substrates of RS2

The accuracy of the molecular docking analysis can vary, and the top-ranked docking pose of an analysis is not always correct. To select the most reliable docking pose, we examined the position and role of W331* in each predicted structure, which has been associated with the RS2 inactivity. Our docking simulation of galactinol against RS2 yielded several poses, but only one had a structure with a hydrogen bond (OH-π) between W331* and the galactose residue of galactinol (Figure 7a), indicating the importance of W331* in ligand binding.

We consider that this docking pose accurately reflects the galactinol–RS2 complex for the following three reasons. First, the right half of the pocket shown in Figure 7a is unoccupied, providing sufficient space for the binding of a disaccharide like sucrose. This pose enables myo-inositol to be released even if the unoccupied pocket space is filled by sucrose. Second, the contact mode of RS2 and galactinol shown in Figure 7b reveals six hydrogen bonds around galactose but no bonds around myo-inositol, indicating that myo-inositol would be more easily released than galactose. Third, the carboxyl group of D474 is located near C1 of galactose, which is a possible reacting point for sucrose. If the reaction mechanism of RS2 is similar to the twice-inverting type described by Schuman et al.,^29^ D474 should be one of the crucial residues for RS2 activity.

This pocket has the potential to bind not only sucrose but also to raffinose. Although the estimated sucrose binding site in this pocket is well-suited for a disaccharide, raffinose could be bound by being partially exposed to the open space; i.e., sucrose could be substituted with raffinose if a myo-inositol and two galactose molecules are positioned correctly in the pocket. This modeled structure suggests that RS2 is a multifunctional enzyme. However, further studies are warranted to verify the mechanisms that regulate the oligosaccharide content.

### GmJMC172 as a Precious Accession with Complete Loss of RS2 Activity without Decreasing Raffinose Content

PI 200508, a soybean landrace with a W331* deletion mutant of RS2, was reported by Dierking and Bilyeu.^15^ In this study, the Alphafold2-predicted 3D structure indicated that W331* is a residue of the ligand-binding pocket. However, in our modeled structure lacking W331*, the whole structure and pocket were similar to the native alternative (data not shown). Therefore, the RS2 protein without W331* does not seem to completely lose RS2 activity. According to previously reported experimental findings on PI200508,^15^ the lack of W331* decreased raffinose content, but complete loss of activity was not directly observed. The strength of a hydrogen bond is several kcal/mol,^30^ so the loss of one hydrogen bond causes a marked decrease in affinity, which is enough to explain the previous experimental observation.^15^ Thus, GmJMC172 may be the only variety with complete loss of RS2 activity, making it a novel soybean accession for progressing future RS2 research.

All these RS2 mutants/varieties were evaluated and a lack/decrease in activity (PI200508, *rs2*, and GmJMC172) caused a reduction of stachyose contents. Among the trails lacking RS2 activity, GmJMC172 does not decrease raffinose content, while the RS2 knockout trait *rs2* has the opposite effect.^27^ A possible reason for this observation could be the tolerance for environmental stress. Change of RFO content due to abiotic stress has been reported for several plant species.^31−33^ Therefore, varieties completely lacking RS could reduce the abiotic tolerance and possess a survival disadvantage. If so, GmJMC172 may have acquired a novel mechanism for raffinose synthesis. We could not detect any unique mutation in the RS3/RS4/STS genes. Hence, mutations that change the expression patterns of the enzymes causing compensation for raffinose synthesis activity may exist in a location other than the structural genes.

Seeds of GmJMC172, as well as GmJMC030 and GmJMC158, are distributed from NARO Genebank (https://www.gene.affrc.go.jp/).

## Abbreviations

3D: three-dimensional
HPLC: high-performance liquid chromatography
NARO: National Agriculture and Food Research Organization
RFO: raffinose family oligosaccharide
RS: raffinose synthase
STS: stachyose synthase
SD: standard deviation
SNP: single-nucleotide polymorphism
